# The Increased Risk for Autoimmune Diseases in Patients with Eating Disorders

**DOI:** 10.1371/journal.pone.0104845

**Published:** 2014-08-22

**Authors:** Anu Raevuori, Jari Haukka, Outi Vaarala, Jaana M. Suvisaari, Mika Gissler, Marjut Grainger, Milla S. Linna, Jaana T. Suokas

**Affiliations:** 1 Hjelt Institute, Department of Public Health, University of Helsinki, Helsinki, Finland; 2 Department of Adolescent Psychiatry, Helsinki University Central Hospital, Helsinki, Finland; 3 Institute of Clinical Medicine, Child Psychiatry, University of Turku, Turku, Finland; 4 Department of Mental Health and Substance Abuse Services, National Institute for Health and Welfare, Helsinki, Finland; 5 Department of Vaccination and Immune Protection, National Institute for Health and Welfare, Helsinki, Finland; 6 Department of Social Psychiatry, Tampere School of Public Health, Tampere, Finland; 7 Information Department, National Institute for Health and Welfare, Helsinki, Finland; 8 Nordic School of Public Health, Gothenburg, Sweden; 9 Department of Psychiatry, Helsinki University Central Hospital, Helsinki, Finland; University of British Columbia, Canada

## Abstract

**Objective:**

Research suggests autoimmune processes to be involved in psychiatric disorders. We aimed to address the prevalence and incidence of autoimmune diseases in a large Finnish patient cohort with anorexia nervosa, bulimia nervosa, and binge eating disorder.

**Methods:**

Patients (N = 2342) treated at the Eating Disorder Unit of Helsinki University Central Hospital between 1995 and 2010 were compared with general population controls (N = 9368) matched for age, sex, and place of residence. Data of 30 autoimmune diseases from the Hospital Discharge Register from 1969 to 2010 were analyzed using conditional and Poisson regression models.

**Results:**

Of patients, 8.9% vs. 5.4% of control individuals had been diagnosed with one or more autoimmune disease (OR 1.7, 95% CI 1.5–2.0, P<0.001). The increase in endocrinological diseases (OR 2.4, 95% CI 1.8–3.2, P<0.001) was explained by type 1 diabetes, whereas Crohn's disease contributed most to the risk of gastroenterological diseases (OR 1.8, 95% CI 1.4–2.5, P<0.001). Higher prevalence of autoimmune diseases among patients with eating disorders was not exclusively due to endocrinological and gastroenterological diseases; when the two categories were excluded, the increase in prevalence was seen in the patients both before the onset of the eating disorder treatment (OR 1.5, 95% CI 1.1–2.1, P = 0.02) and at the end of the follow-up (OR 1.4, 95% CI 1.1–1.8, P = 0.01).

**Conclusions:**

We observed an association between eating disorders and several autoimmune diseases with different genetic backgrounds. Our findings support the link between immune-mediated mechanisms and development of eating disorders. Future studies are needed to further explore the risk of autoimmune diseases and immunological mechanisms in individuals with eating disorders and their family members.

## Introduction

Eating disorders are relatively common multifactorial disorders, the etiology of which appears to be regulated by interplay of environmental and genetic factors. They are often associated with substantial somatic morbidity [1]–[2], since psychological stress combined with dysregulated eating behavior and subsequent nutritional disturbances have a potent effect on several organ systems. On the other hand, the risk of eating disorders has been shown to be increased in some somatic illnesses [3]–[5]. Notably, many of these illnesses, such as type 1 diabetes (T1D) and inflammatory bowel diseases present autoimmune or autoinflammatory etiology.

A prior autoimmune disease has recently been shown to increase the risk of mood disorders and schizophrenia [6]–[7]. In addition, the risk of both mental disorders increased in a dose response pattern when autoimmune diseases and infections were assessed together. The role of autoimmune processes, such as various pathogens triggering autoantibodies cross-reactive with neuronal antigens (brain-reactive autoantibodies), has also been recognized in the pathogenesis of neuropsychiatric disorders [8] including autism spectrum disorders, obsessive-compulsive disorder, tic-disorders, ADHD, post-traumatic stress disorder, and narcolepsy. Furthermore, pediatric Autoimmune Neuropsychiatric Disorders Associated with Streptococcal infection (PANDAS) include anorexia nervosa (AN) [8].

As evidence that autoimmune mechanisms, acting via neuroimmunoendocrinological pathways, could be involved in eating disorders, Fetissov et al. [9] reported that a significant subset of patients with AN and bulimia nervosa (BN) had autoantibodies against α-melanocyte-stimulating hormone (α-MSH) and against adrenocorticotropic hormone (ACTH), which contribute to food intake and body weight. The authors [10] subsequently observed that the core psycho-behavioral characteristics of eating disorders correlated with the levels of autoantibodies against α-MSH; notably, these correlations were opposite in patients with AN vs. BN. In an independent sample, decreased levels of immunoglobulins G, M, and A classes of autoantibodies reacting with acyl ghrelin in AN were also reported [11].

Immune-mediated diseases often show an association with polymorphisms of genes regulating the function of immune system. Human leukocyte antigen (HLA) region in chromosome 6 embodies genes, which have been associated with several autoimmune diseases. Despite genetic factors appearing important in the etiology of eating disorders, reports of HLA gene polymorphisms are contradictory. In one study, a higher frequency of HLA-Bw16 was reported in patients with AN [12], but the observation was not supported by later reports in AN [13], or in BN [14].

Prevalence of eating disorders in T1D is reported two-fold among adolescents in a recent meta-analysis [15]. Utilizing the current sample, we recently reported a vastly increased T1D-related mortality in eating disorders [16]. However, studies of the risk of T1D in individuals with eating disorders are, according to our knowledge, lacking. Abnormal thyroid values occur in eating disorders [17], and starvation as well as re-feeding are associated with altered thyroid function [18]. Crohn's disease and celiac disease have been suggested to act as triggers for the development of eating pathology [19]–[20], and individuals with celiac disease are reported to be at increased risk for eating disorders [3]. Unsurprisingly, inflammation of intestinal mucosa may affect eating patterns; mouse models imply resultant reduced food intake, where eating is under a specific control of CD4+ T-cells [21]. Finally, increased prevalence of metabolic syndrome and eating disorders in individuals with psoriasis has been reported [22]; the pathogenic mechanism is proposed to relate to immune-mediated inflammation.

To our knowledge, no large scale reports of the co-morbidity of autoimmune diseases and eating disorders have been published. We hypothesized that the risk of autoimmune diseases would be increased in individuals with eating disorders both prior and after the onset of the treatment for an eating disorder. Thus, we aimed to address the incidence and prevalence of autoimmune diseases in a large Finnish patient cohort of individuals treated for AN, BN, and binge eating disorder (BED) during 16 years' period. We explored the potential associations in three stages; period prevalence at the onset of the treatment of an eating disorders, accounting for both prevalent and new onset autoimmune disease cases for the time before the treatment; incidence after the treatment onset until the end of the study period to yield the information of new onset autoimmune diseases and to separate the potential improved effect of the treatment system to detect the autoimmune diseases among patients compared to controls (detection bias); and finally, lifetime prevalence including prevalent and new onset autoimmune disease cases any time before, during, or after the treatment until the end of the follow-up.

## Materials and Methods

### Ethical considerations

Ethical permits were obtained from the Ethics committee of National Institute of Health and Welfare (Dnro THL/184/6.02.00/2011). The study was conducted according to the Helsinki Declaration. Data handling was performed according to the data protection legislation and the rules of National Institute of Health and Welfare. All register keeping institutions gave permission to use their data in scientific research. The authors did not have access to personal identification data, only research codes were used in analyses.

### Study participants

We identified all patients (N = 2342) treated in the Eating Disorder Unit at the Helsinki University Central Hospital from January 1^st^ 1995 to December 30^st^ 2010. For each patient, four controls (N = 9368) were selected from the Central Population Register, and matched for age, sex, and place of residence. The individual follow-up period for incidence of each patient and the controls started at treatment in the Unit and ended at the first autoimmune disease hospitalization, or December 31^st^ 2010, or death (N = 61 patients, N = 69 controls). Only individuals without autoimmune disease before the start of follow-up were included in the incidence calculations. We calculated period prevalence of hospital-treated autoimmune diseases starting at January 1^st^ 1969 until the date of the start of the eating disorder treatment. At the end of the follow-up, lifetime prevalence was calculated utilizing Hospital Discharge Register data from January 1^st^ 1969 to December 31^st^ 2010.

Eating disorder diagnosis was set by the attending psychiatrist at the Eating Disorder Unit on the patient's arrival using the ICD-10 criteria [23]. ICD-9 diagnoses given in 1995 were re-coded to ICD-10 codes. The participants included patients with ICD-10 diagnoses of F50.0, F50.1, F50.2, F50.3, and F50.8 indicating AN, atypical AN, BN, atypical BN, and BED, respectively. Since current definitions for eating disorders in DSM-5 [24] have been relaxed compared to ICD-10 or DSM-IV criteria, we defined AN and BN as follows: AN included AN and atypical AN. Atypical AN diagnosis was used for patients in whom some key features of AN, such as amenorrhea or body weight less than 85% of that expected, were lacking but who otherwise presented a typical clinical picture. BN included BN and atypical BN. Atypical BN diagnosis was used in patients among whom one or two criteria were lacking, but who otherwise presented a typical clinical picture. BED, coded as F50.8, ‘Other eating disorder’, was diagnosed in the Eating Disorder Unit according to the DSM-IV research criteria.

### Assessment of autoimmune disease

The study population was linked to the data of 30 autoimmune diseases (Table S1) obtained from the Hospital Discharge Register. It covers all public/private general and psychiatric hospitals, hospital outpatient clinics and health center inpatient wards. Diagnoses were made by the attending physician. The registry contains hospital identification codes, dates of stay and primary diagnoses at discharge with up to three subsidiary diagnoses.

Time of the onset of an autoimmune diagnosis was defined as the first day of the hospital contact leading to the recording in the Hospital Discharge Register. Each participant could have more than one autoimmune disease. From 1969 to 1986, ICD-8 was used as the diagnostic classification, between 1987 and 1995 it was ICD-9, and since 1996, it was ICD-10.

### Statistical analyses

Incidence was analyzed using Poisson regression model where occurrence of autoimmune disease was used as an outcome variable and logarithm of cumulated person-years as an offset term. Results are reported as rate ratios (RR) with 95% confidence intervals (CI). Period and lifetime prevalences were modeled using conditional logistic regression model, conditioned with a matching group; odds ratios (OR) with 95% CI are reported. All calculations were carried out by R language [25].

## Results

### General

Characteristics of the study sample are presented in the Table 1. Participants with BN made up the largest patient group (54.0%), followed by those with AN (38.8%), and those with BED (7.3%). The mean age of patients with eating disorders at the start of the follow-up differed significantly (P<0.001) between diagnostic groups, patients with AN being the youngest, and those with BED the oldest. Within eating disorder categories, proportion of males ranged from 2.3% in BN to 12.9% in BED, being 4.8% in AN (P<0.001).

**Table 1:** Study sample by diagnostic groups with lifetime prevalence figures of autoimmune diseases covering the time before, during, and after the treatment for an eating disorder.

		Anorexia Nervosa (N = 911)	Bulimia Nervosa (N = 1260)	Binge Eating Disorder (N = 171)
		F50.0 Anorexia Nervosa F50.1 Atypical Anorexia Nervosa	F50.2 Bulimia Nervosa F50.3 Atypical Bulimia Nervosa	F50.8 Other Eating Disorder, DSM-IV BED
**Mean age, years (SD, Range)**		23.8 (6.8, 16.0–64.8)	26.2 (7.5, 17.2–64.6)	37.0 (10.6, 17.8–64.2)
**Total number participants with ED (% within a diagnostic group)**	**Women**	867	1231	149
	**Men**	44 (4.8%)	29 (2.3%)	22 (12.9%)
**Number (%) of participants ever treated as inpatient for ED**	**Women & men**	322 (35.3%)	134 (10.6%)	7 (4%)
**Participants with ED & with any autoimmune disease (N, positive/negative for the autoimmune disease, % positive)**	**Women**	60/807 (6.9%)	111/1120 (9.0%)	26/123 (17.4%)
	**Men**	4/40 (9.1%)	7/22 (24.1%)	2/20 (9.1%)
	**Total**	64/847 (7.0%)	118/1142 (9.4%)	27/144 (15.8%)
**Matched control participants with any autoimmune disease (N, positive/negative for the autoimmune disease, % positive)**	**Women**	166/3302 (4.8%)	288/4636 (5.8%)	36/560 (6.0%)
	**Men**	5/171 (2.8%)	8/108 (6.9%)	6/82 (6.8%)
	**Total**	171/3473 (4.7%)	296/4744 (5.8%)	42/642 (6.1%)

Each participant is presented as one observation, i.e. some autoimmune disease-positive individuals had more than one autoimmune disease. ED, Eating Disorder.

### Period prevalence of autoimmune diseases until the start of the follow-up

Until the start of the treatment for an eating disorder, 5.6% (N = 131) of patients and 2.8% of control individuals (N = 265) had been diagnosed with one or more autoimmune disease (Table 2). The risk of prior diagnosis of endocrinological autoimmune diseases (OR 3.3, 95% CI 2.4–4.6, P<0.001), of gastroenterological immune-mediated diseases (OR 2.0, 95% CI 1.3–3.1, P = 0.002), and of autoimmune diseases combined (OR 2.1 95% CI 1.7–2.7, P<0.001) was significantly higher among patients than among matched controls. This was not solely due to endocrinological and gastroenterological diseases; when we excluded these two categories and analyzed other categories combined, the difference between the patients and controls remained (OR 1.5, 95% CI 1.1–2.1, P = 0.02).

**Table 2:** Period prevalences and results from conditional logistic regression models of autoimmune diseases from the start of the study period until the beginning of the treatment of an eating disorder.

Autoimmune disease category	N (Positive/negative for the autoimmune disease, % positive)	OR (95% CI)	P-value
***Any autoimmune disease***			
**Controls**	265/9103 (2.8%)	reference	-
**All eating disorders**	131/2211 (5.6%)	2.13 (1.71–2.65)	<0.001
**Anorexia Nervosa**	40/871 (4.4%)	1.76 (1.20–2.59)	0.004
**Bulimia Nervosa**	71/1189 (5.6%)	2.18 (1.62–2.93)	<0.001
**Binge eating Disorder**	20/151 (11.7%)	2.79 (1.54–5.03)	<0.001
***Endocrinological diseases***			
**Controls**	83/9285 (0.89%)	reference	-
**All eating disorders**	65/2277 (2.78%)	3.28 (2.35–4.59)	<0.001
**Anorexia Nervosa**	16/895 (1.76%)	1.95 (1.05–3.59)	0.03
**Bulimia Nervosa**	41/1219 (3.25%)	4.35 (2.77–6.82)	<0.001
**Binge Eating Disorder**	8/163 (4.68%)	3.56 (1.37–9.22)	0.009
***Gastroenterological diseases***			
**Controls**	61/9307 (0.65%)	reference	-
**All eating disorders**	30/2312 (1.28%)	1.98 (1.27–3.07)	0.002
**Anorexia Nervosa**	11/900 (1.21%)	2.32 (1.10–4.87)	0.03
**Bulimia Nervosa**	15/1245 (1.19%)	1.59 (0.87–2.90)	0.13
**Binge Eating Disorder**	4/167 (2.34%)	4.00 (1.00–16.0)	0.05
***Ocular diseases***			
**Controls**	20/9238 (0.21%)	reference	-
**All eating disorders**	8/2334 (0.34%)	1.62 (0.71–3.71)	0.26
**Anorexia Nervosa**	1/910 (0.11%)	0.80 (0.09–6.85)	0.84
**Bulimia Nervosa**	4/1256 (0.32%)	1.15 (0.37–3.55)	0.81
**Binge Eating Disorder**	3/168 (1.75%)	12.0 (1.25–115.0)	0.03
***Dermatological diseases***			
**Controls**	23/9345 (0.25%)	reference	-
**All eating disorders**	7/2335 (0.30%)	1.22 (0.52–2.84)	0.65
**Anorexia Nervosa**	2/909 (0.22%)	0.89 (0.19–4.11)	0.88
**Bulimia Nervosa**	3/1257 (0.24%)	1.00 (0.28–3.54)	1.00
**Binge Eating Disorder**	2/169 (1.17%)	4.00 (0.56–28.4)	0.17
***Connective tissue diseases***			
**Controls**	60/9308 (0.64%)	reference	-
**All eating disorders**	22/2320 (0.94%)	1.47 (0.90–2.39)	0.12
**Anorexia Nervosa**	9/902 (0.99%)	1.71 (0.79–3.74)	0.18
**Bulimia Nervosa**	6/1254 (0.48%)	0.92 (0.38–2.24)	0.86
**Binge Eating Disorder**	7/164 (4.09%)	2.15 (0.86–5.40)	0.10
***Neurological diseases***			
**Controls**	17/9351 (0.18%)	reference	-
**All eating disorders**	4/2338 (0.17%)	0.94 (0.32–2.80)	0.91
**Anorexia Nervosa**	1/910 (0.11%)	1.33 (0.14–12.8)	0.8
**Bulimia Nervosa**	3/1257 (0.24%)	1.00 (0.28–3.54)	1.00
**Binge Eating Disorder**	0/171	-	-
***Hematological diseases***			
**Controls**	7/9361 (0.07%)	reference	-
**All eating disorders**	3/2339 (0.13%)	1.71 (0.44–6.63)	0.43
**Anorexia Nervosa**	2/909 (0.22%)	4.00 (0.56–28.4)	0.17
**Bulimia Nervosa**	1/1259 (0.08%)	0.8 (0.09–6.85)	0.84
**Binge Eating Disorder**	0/171	-	-

For the seven separate autoimmune disease categories presented, each participant counts as maximum one observation per category. For the total figure of any autoimmune disease, each participant is presented as maximum one observation. Out of all patients and all controls, there was only one patient participant (with bulimia nervosa) with pulmonary disease (not shown). CI, Confidence Interval; OR, Odds ratio.

The higher period prevalence of endocrinological disease was explained by T1D (2.5% in patients *vs*. 0.6% in controls; OR 4.3, 95% CI 3.0–6.3), not by thyreotoxicosis (OR 1.3, 95% CI 0.5–1.7) or autoimmune thyroiditis (OR 1.1, 95% CI 0.2–5.5). When analyzed separately in AN, BN and BED, the period prevalence of endocrinological diseases was increased (OR 2.0, 95% CI 1.1–3.6; OR 4.4, 95% CI 2.8–6.8; OR 3.6, 95% CI 1.4–9.2, respectively). The increased prevalence of gastroenterological diseases among patients was largely explained by Crohn's disease (0.6% in patients *vs*. 0.2% in controls; OR 3.1, 95% CI 1.5–6.3), not by celiac disease (OR 1.4, 95% CI 0.7–3.1), or ulcerative colitis (OR 1.6, 95% CI 0.7–3.2). When analyzed separately in AN, BN and BED, the period prevalence of gastroenterological diseases was significantly increased in AN (OR 2.3, 95% CI 1.1–4.9), but not in BN (OR 1.6, 95% CI 0.9–2.9) or BED (OR 4.0, 95% CI 1.0–16.0, P = 0.05).

### Incidence of autoimmune diseases after the start of the follow-up

We examined the incidence of new onset autoimmune diseases beginning at the day that the participants entered to the treatment for an eating disorder until the end of the study period (Table 3). After the start of the treatment, a total of 78 new onset autoimmune disease cases were observed in patients, while 244 new onset cases were observed in control individuals (RR 1.2, 95% CI 0.9–1.5, P = 0.26). The incidence of gastroenterological diseases and of dermatological diseases were increased in patients with eating disorders (RR 1.7, 95% CI 1.1–2.6, and RR 1.8, 95% CI 1.0–3.2, respectively), and separately in BN (RR 1.9, 95% CI 1.2–3.2, and RR 2.2 95% CI 1.2–4.1). The incidences of ocular and connective tissue diseases were increased in BED (RR 4.5, 95% CI 1.4–14.6 and RR 3.7, 95% CI 1.2–11.9). We did not find evidence for differences in the evolution of incidence rate curves of a composite of all autoimmune diseases between patients and controls (for hazard curves, see Figure S1), i.e. there was no evidence for detection bias.

**Table 3:** Incidence of new onset autoimmune diseases after the start of the treatment for an eating disorder until the end of the study period.

Autoimmune disease category	N Total	N Events	Person-years (1/1000)	Incidence (95% CI)	RR (95% CI)
***Any autoimmune disease***					
**Controls**	9103	244	87.264	3.12 (2.76–3.52)	reference
**All eating disorders**	2211	78	21.242	3.62 (2.86–4.53)	1.16 (0.90–1.49)
**Anorexia nervosa**	871	24	7.285	3.29 (2.11–4.90)	1.05 (0.69–1.60)
**Bulimia Nervosa**	1189	47	12.634	3.56 (2.59–4.77)	1.14 (0.83–1.56)
**Binge Eating Disorder**	151	7	1.323	6.05 (2.61–11.92)	1.93 (0.96–3.90)
***Endocrinological diseases***					
**Controls**	9285	56	83.262	0.67 (0.51–0.87)	reference
**All eating disorders**	2277	16	20.253	0.79 (0.45–1.28)	1.17 (0.67–2.05)
**Anorexia nervosa**	895	4	6.999	0.57 (0.16–1.46)	0.85 (0.31–2.43)
**Bulimia Nervosa**	1219	11	12.055	0.91 (0.46–1.63)	1.36 (0.71–2.59)
**Binge Eating Disorder**	163	1	1.196	0.84 (0.02–4.66)	1.24 (0.17–8.98)
***Gastroenterological diseases***					
**Controls**	9307	69	83.677	0.83 (0.64–1.04)	reference
**All eating disorders**	2312	29	20.872	1.39 (0.93–2.00)	1.68 (1.09–2.60)*
**Anorexia nervosa**	900	9	7.068	1.27 (0.58–2.42)	1.54 (0.77–3.09)
**Bulimia Nervosa**	1245	20	12.608	1.59 (0.97–2.45)	1.92 (1.17–3.16)*
**Binge Eating Disorder**	167	0	1.198	0 (0–3.1)	0
***Ocular diseases***					
**Controls**	9348	43	83.517	0.51 (0.37–0.69)	reference
**All eating disorders**	2334	16	20.710	0.77 (0.44–1.25)	1.50 (0.85–2.66)
**Anorexia nervosa**	910	6	7.102	0.84 (0.31–1.84)	1.64 (0.70–3.85)
**Bulimia Nervosa**	1256	7	12.322	0.57 (0.22–1.17)	1.10 (0.50–2.45)
**Binge Eating Disorder**	168	3	1.283	2.34 (0.48–6.83)	4.54 (1.41–14.64)*
***Dermatological diseases***					
**Controls**	9345	40	83.410	0.48 (0.34–0.65)	reference
**All eating disorders**	2335	18	20.720	0.87 (0.51–1.37)	1.81 (1.04–3.16)*
**Anorexia nervosa**	909	3	7.006	0.43 (0.09–1.25)	0.89 (0.28–2.89)
**Bulimia Nervosa**	1257	13	12.440	1.05 (0.56–1.79)	2.18 (1.17–4.07)*
**Binge Eating Disorder**	169	2	1.275	1.57 (0.19–5.67)	3.27 (0.79–13.53)
***Connective tissue diseases***					
**Controls**	9308	53	83.507	0.63 (0.48–0.83)	reference
**All eating disorders**	2320	17	20.650	0.82 (0.48–1.32)	1.30 (0.75–2.24)
**Anorexia nervosa**	902	5	7.065	0.71 (0.23–1.65)	1.12 (0.45–2.79)
**Bulimia Nervosa**	1254	9	12.312	0.73 (0.33–1.39)	1.16 (0.57–2.33)
**Binge Eating Disorder**	164	3	1.273	2.36 (0.49–6.89)	3.71 (1.16–11.88)*
***Neurological diseases***					
**Controls**	9351	19	82.858	0.23 (0.14–0.36)	reference
**All eating disorders**	2338	6	20.450	0.29 (0.11–0.64)	1.28 (0.51–3.20)
**Anorexia nervosa**	910	4	7.056	0.57 (0.15–1.45)	2.47 (0.84–7.27)
**Bulimia Nervosa**	1257	2	12.175	0.16 (0.02–0.59)	0.72 (0.17–3.08)
**Binge Eating Disorder**	171	0	1.221	0 (0–3.02)	0
***Hematological diseases***					
**Controls**	9361	3	82.5654	0.04 (0.008–0.11)	reference
**All eating disorders**	2339	0	20.331	0	-
**Anorexia nervosa**	909	0	6.947	0	-
**Bulimia Nervosa**	1259	0	12.163	0	-
**Binge Eating Disorder**	171	0	1.221	0	-

Each participant accounted as maximum one observation per autoimmune disease category (according to the first incident autoimmune disease).Those who already had had an autoimmune disease (Table 2) included in each category, were excluded. In the section of ‘*Any autoimmune disease*’**,** those who had had any previous autoimmune disease were excluded. There were no incident cases in pulmonary diseases. CI, Confidence Interval; RR, Risk Ratio.

*Statistically significant P-values.

### Lifetime prevalence of autoimmune diseases at the end of the follow-up

In the whole study period, 8.9% (N = 209) of patients and 5.4% of control individuals (N = 509) had ever been diagnosed with one or more autoimmune disease (OR 1.7, 95% CI 1.5–2.0, P<0.001) (Table 4). Of patients with an eating disorders and with at least one autoimmune disease, 21% (N = 44) had more than one autoimmune disease, while of controls, the proportion was 15% (N = 78) (P = 0.08). Among patients with BN, the lifetime prevalence of autoimmune diseases was higher in men compared to women (24% *vs*. 9%, P = 0.006). In other eating disorders or in control groups, no significant sex-differences were observed.

**Table 4:** Lifetime prevalences of autoimmune diseases, i.e. status at the end of the study period covering the time before, during and after the treatment for an eating disorder in patients with eating disorders compared with their control individuals.

Autoimmune disease category	N (Positive/negative for the autoimmune disease, % positive)		OR (95% CI)	P-value
	Patients	Controls		
***Any autoimmune disease***				
**All eating disorders**	209/2133 (8.9%)	509/8859 (5.4%)	1.7 (1.5–2.0)	<0.001
**Anorexia Nervosa**	64/847 (7.0%)	171/3473 (4.7%)	1.5 (1.1–2.1)	0.004
**Bulimia Nervosa**	118/1142 (9.4%)	296/4744 (5.9%)	1.7 (1.3–2.1)	<0.001
**Binge Eating Disorder**	27/144 (15.8%)	42/642 (6.1%)	2.8 (1.7–4.7)	<0.001
***Endocrinological diseases***				
**All eating disorders**	81/2261 (3.5%)	139/9229 (1.5%)	2.4 (1.8–3.2)	<0.001
**Anorexia Nervosa**	20/891 (2.2%)	57/3587 (1.6%)	1.4 (0.8–2.3)	0.2
**Bulimia Nervosa**	52/1208 (4.1%)	71/4969 (1.4%)	3.1 (2.1–4.5)	<0.001
**Binge Eating Disorder**	9/162 (5.3%)	11/673 (1.6%)	3.3 (1.4–7.9)	0.008
***Gastroenterological diseases***				
**All eating disorders**	59/2283 (2.5%)	115/9253 (1.2%)	1.8 (1.4–2.5)	<0.001
**Anorexia Nervosa**	20/891(2.2%)	46/3598 (1.3%)	1.8 (1.03–3.0)	0.04
**Bulimia Nervosa**	35/1225 (2.8%)	79/4961 (1.6%)	1.8 (1.2–2.7)	0.004
**Binge Eating Disorder**	4/167 (2.3%)	5/679 (0.7%)	3.2 (0.86–11.9)	0.08
***Ocular diseases***				
**All eating disorders**	24/2318 (1.0%)	63/9305 (0.7%)	1.5 (0.96–2.5)	0.08
**Anorexia Nervosa**	7/904 (0.8%)	17/3627 (0.5%)	1.7 (0.7–4.0)	0.3
**Bulimia Nervosa**	11/1249 (0.9%)	44/4996 (0.9%)	1.0 (0.5–2.0)	1.0
**Binge Eating Disorder**	6/165 (3.5%)	2/682 (0.3%)	12 (2.4–59.5)	0.002
***Dermatological diseases***				
**All eating disorders**	25/2317 (1.1%)	63/9305 (0.7%)	1.6 (1.0–2.52)	0.05
**Anorexia Nervosa**	5/906(0.5%)	16/3628 (0.4%)	1.3 (0.5–3.4)	0.7
**Bulimia Nervosa**	16/1244(1.3%)	43/4997 (0.9%)	1.5 (0.8–2.6)	0.17
**Binge Eating Disorder**	4/167(2.3%)	4/680 (0.6%)	4.0 (1.0–16.0)	0.05
***Connective tissue diseases***				
**All eating disorders**	39/2303 (1.7%)	113/9255 (1.2%)	1.4 (0.96–2.0)	0.08
**Anorexia Nervosa**	14/897 (1.5%)	30/3614 (0.8%)	1.9 (1.0–3.5)	0.05
**Bulimia Nervosa**	15/1245(1.2%)	61/4979 (1.2%)	1.0 (0.5–1.8)	0.9
**Binge Eating Disorder**	10/161 (5.8%)	22/662 (3.2%)	1.8 (0.8–4.0)	0.11
***Neurological diseases***				
**All eating disorders**	10/2332 (0.4%)	36/9332 (0.4%)	1.1 (0.5–2.2)	0.8
**Anorexia Nervosa**	5/906 (%)	10/3634 (0.3%)	2.0 (0.7–5.9)	0.2
**Bulimia Nervosa**	5/1255 (0.4%)	24/5016 (%)	0.8 (0.3–2.2)	0.7
**Binge Eating Disorder**	0/171	2/682 (0.3%)	-	-
***Hematological diseases***				
**All eating disorders**	3/2339 (0.1%)	10/9358 (0.1%)	1.2 (0.3–4.4)	0.78
**Anorexia Nervosa**	2/909 (0.2%)	2/3642 (0.05%)	4.0 (0.6–28.4)	0.17
**Bulimia Nervosa**	1/1259 (0.08%)	8/5032 (0.2%)	0.5 (0.1–4.0)	0.5
**Binge Eating Disorder**	0/171	0/684	-	-
***Pulmonary diseases***				
**All eating disorders**	1/2341 (0.04%)	0/9368	-	-
**Anorexia Nervosa**	0/911	0/3644	-	-
**Bulimia Nervosa**	1/1259	0/5040	-	-
**Binge Eating Disorder**	0/171	0/684	-	-

The lifetime prevalence of endocrinological diseases was increased in eating disorders (OR 2.4, 95% CI 1.8–3.2, P<0.001), and when analyzed separately in BN (OR 3.1, 95% CI 2.1–4.5, P<0.001) and in BED (OR 3.3, 95% CI 1.4–7.9, P = 0.008) (Table 4). The finding was explained by increase in lifetime prevalence of T1D (2.9% in patients *vs*. 0.8% in controls, OR 3.8, 95% CI 2.7–5.3, P<0.001) (Table 5 and Table S2). The lifetime prevalence of gastroenterological diseases was increased in eating disorders combined (OR 1.8, 95% CI 1.4–2.5, P<0.001) and in AN (OR 1.8, 95% CI 1.03–3.0, P = 0.04) and BN (OR 1.8, 95% CI 1.2–2.7, P = 0.004) separately. The finding was largely explained by the increased prevalence of Crohn's disease (1.1% in patients *vs*. 0.4% in controls, OR 3.1, 95% CI 1.9–5.1, P<0.001) (Table 5). Also, the lifetime prevalence was increased for ocular disorders (OR 12.0, 95% CI 2.4–59.5, P = 0.002) in BED. By disentangling individual autoimmune diseases (Table 5 and Table S2), the risk of systemic lupus erythematotus (SLE) was increased in eating disorders (0.3% in patients *vs*. 0.06% in controls, OR 4.0, 95% CI 1.3–12.4, P = 0.02).

**Table 5:** Autoimmune disease status per each individual autoimmune disease included in the study at the end of the study period (covering the time before, during and after the treatment) for a composite of all patients with eating disorder compared with their control individuals.

Autoimmune disease category	N (Positive/negative for the autoimmune disorder, % positive)		OR (95% CI)	P-value
	Patients	Controls		
***Endocrinological diseases***				
**Type I diabetes mellitus**	68/2274 (2.9%)	76/9292 (0.8%)	3.79 (2.70–5.31)	<0.001
**Autoimmune hyperthyroidism (Basedow's disease, Grave's disease)**	13/2329 (0.6%)	56/9312 (0.6%)	0.93 (0.51–1.7)	0.81
**Autoimmune thyroiditis (Hashimoto's disease)**	2/2340 (0.09%)	9/9359 (0.1%)	0.89 (0.19–4.11)	0.88
**Adrenal insufficiency (Addison's disease)**	1/2341 (0.04%)	0/9368	-	-
***Gastroenterological diseases***				
**Celiac disease**	16/2326 (0.7%)	44/9324 (0.5%)	1.45 (0.82–2.58)	0.2
**Regional enteritis (Crohn's disease)**	27/2315 (1.1%)	35/9333 (0.4%)	3.09 (1.87–5.10)	<0.001
**Ulcerative colitis**	24/2318 (1.0%)	69/9299 (0.7%)	1.4 (0.88–2.24)	0.16
**Primary biliary cirrhosis**	0/2342	2/9366 (0.02%)	-	-
***Ocular diseases***				
**Iridocyclitis**	24/2318 (1.0%)	63/9305 (0.7%)	1.54 (0.96–2.47)	0.08
***Dermatological diseases***				
**Pemphigus/pemphigoid**	1/2341 (0.04%)	3/9365 (0.03%)	1.33 (0.14–12.8)	0.8
**Dermatitis herpetiformis (skin condition associated with celiac disease)**	0/2342	0/9368	-	-
**Psoriasis**	22/2320 (0.9%)	56/9312 (0.6%)	1.57 (0.96–2.57)	0.07
**Vitiligo**	1/2341 (0.04%)	3/9365 (0.03%)	1.33 (0.14–12.8)	0.8
**Lupus Erythematosus Discoides (LED)**	1/2342 (0.04%)	1/9367 (0.01%)	4.0 (0.25–64.0)	0.33
***Connective tissue diseases***				
**Rheumatoid arthritis**	15/2327 (0.6%)	45/9323 (0.5%)	1.33 (0.74–2.39)	0.33
**Arthritis ** ***rheumatoides juvenilis***	11/2331 (0.5%)	34/9334 (0.4%)	1.29 (0.66–2.55)	0.46
**Ankylosing spondylitis**	7/2335 (0.3%)	30/9338 (0.3%)	0.93 (0.41–2.12)	0.87
**Polymyositis/Dermatomyositis**	1/2341 (0.04%)	4/9364 (0.04%)	1.0 (0.11–8.95)	1.00
**Systemic lupus erythematosus (SLE)**	6/2336 (0.3%)	6/9362 (0.06%)	4.0 (1.29–12.4)	0.02
**Systemic scleroderma**	1/2341 (0.04%)	2/9366 (0.02%)	2.0 (0.18–22.1)	0.57
**Mixed Connective Tissue Disease (MCTD)**	2/2340 (0.09%)	2/9366 (0.02%)	4.0 (0.56–28.4)	0.17
**Sjögren's syndrome**	9/2333 (0.4%)	17/9351 (0.2%)	2.12 (0.94–4.75)	0.07
**Sarcoidosis**	0/2342	2/9366 (0.02%)	-	-
***Vasculitides***	2/2340 (0.09%)	4/9364 (0.04%)	2.0 (0.37–10.90)	0.42
***Neurological diseases***				
**Multiple sclerosis**	8/2334 (0.3%)	31/9337 (0.3%)	1.03 (0.48–2.25)	0.94
**Myasthenia gravis**	2/2340	5/9363 (0.05%)	1.6 (0.31–8.25)	0.57
***Hematological diseases***				
**Autoimmune hemolytic anemia**	2/2340 (0.09%)	1/9367 (0.01%)	8.0 (0.73–88.20)	0.09
**Idiopathic thrombocytopenic purpura (ITP)**	0	6/9362 (0.06%)	-	-
**Pernicious anemia**	1/2341 (0.04%)	3/9365 (0.03%)	1.33 (0.14–12.8)	0.8
***Pulmonary diseases***				
**Idiopathic fibrosing alveolitis**	1/2341 (0.04%)	0/9368	-	-

Odds Ratios (OR) are presented if meaningful analyses were possible.

Again, the higher lifetime prevalence of autoimmune diseases among patients was not solely due to higher prevalences in endocrinological and gastroenterological diseases; when we excluded these two categories and analyzed the prevalence of all other autoimmune diseases combined, the difference between patients with ED and control individuals remained (OR 1.4, 95% CI 1.1–1.8, P = 0.01).

Finally, we analyzed combined lifetime prevalence of autoimmune diseases with suspected presence of brain-reactive antibodies (autoimmune thyroiditis, celiac disease, multiple sclerosis, Sjögren's syndrome, SLE, thyreotoxicosis, T1D) and of all other autoimmune diseases. OR for the former group was 2.0 (95% CI 1.6–2.5, P<0.0001), and for others it was 1.6 (95% CI 1.4–1.9, P<0.0001).

## Discussion

We observed an increased risk for several autoimmune diseases among patients with eating disorders supporting the hypothesis of co-morbidity of these disorders and suggesting that immune-mediated mechanisms could play a role in the development of eating disorders. Importantly, our results were not restricted to the association of T1D with eating disorders as shown in previous studies. Instead, the association was seen for several autoimmune diseases with different genetic backgrounds. Our findings thus suggest that the link between eating disorders and autoimmune diseases is based on shared immunological mechanisms, rather than on the shared genetic background, e.g. the shared HLA risk genotype. In addition, our findings support earlier observations suggesting that autoimmune processes contribute to the onset and maintenance of eating disorders, at least in a subpopulation of patients.

Studies indicate that psychiatric disorders co-exist with inflammation, infections and autoimmune diseases [6]–[7], [26]–[27], and shared vulnerability [28]–[29] underlying many psychiatric disorders suggest that findings from one disorder may be relevant across categories. Pro-inflammatory cytokines and antibodies/autoantibodies against neuronal antigens could induce changes in neurotransmitter and neuroendocrine function, which may subsequently yield psychiatric manifestations. Studies suggest that pro-inflammatory cytokines may have a role in eating disorders [30]. However, findings on the circulating cytokines, of which many decrease food intake, are discrepant. In most longitudinal studies in AN and BN, their levels return to normal after re-feeding [30], yet it remains unclear whether the alterations in the cytokines are due to an eating disorders or due to an underlying exposure leading to eating disorders. It has also been suggested [30] that pro-inflammatory cytokines might be overproduced in specific brain areas and act locally without concomitant increase in serum or immune production. Indeed, the pro-inflammatory cytokines are able to activate HPA axis [31], the hyperactivity of which in eating disorders has been established [32].

Brain-reactive antibodies can be divided to those with direct effect on the development of the neuropsychiatric symptoms, those generated secondary to the disease process, and those without known association with symptoms [33]. Interestingly, a stronger association with mood disorders was reported among patients with autoimmune diseases with a suspected presence of brain-reactive antibodies compared to the patients with other autoimmune diseases [6]. In our study, there was no significant difference in the strength of the association between the two groups of autoimmune diseases. However, unlike in mood disorders, other than primarily brain-centered mechanisms may more readily be hypothesized to be involved in the pathogenesis of eating disorders. Chief candidates include gastrointestinal peptides influencing visceral organs and central and peripheral nervous systems.

Our findings are supported by the immunological studies performed in patients with eating disorders [9]–[11], [34], where autoantibodies against peptides related to appetite-regulation, stress response, and social-emotional functioning (α-MSH, ACTH, ghrelin, oxytocin, vasopressin) were detected. The postulated role of intestinal microflora contributing to the development of cross-reactive neuronal autoantibodies provides a link between gut and brain. This link may be particularly relevant in eating disorders [34] based on the pivotal role of nutrition in the composition of the gut microbiome. In eating disorders, the core symptom of disordered eating, via deviant food intake and emotional stress, impacts microbial homeostasis and modifies the composition of the intestinal microflora [34]. In addition, purging behavior, such as laxative abuse, may further damage intestinal mucosa and affect gut permeability. In line, 11 new bacterial species, and 19 species never isolated from human gut before were recently identified from a stool sample of an AN patient [35]. Another study of the gut bacterial composition in patients with AN revealed an increase in methanogen M. smithii, which was suggested to be due to an adaptive use of nutrients [36]. Not surprisingly, microbiome of AN patients diverged from that of normal weight and obese population. Gut microbiota is an important regulator of the immune system and its alteration has been associated with autoimmune diseases and immune-mediated disorders, such as allergies and T1D [37]. Composition of the microbiota affects gut permeability, and the function of both innate and adaptive immune system including development of regulatory T-cells [37]. Finally, sex hormones modulate microbiota and the development of autoimmunity [38], as well as the eating disorders risk [39]. The interplay between gut microbiome, immune regulation, and sex hormones thus provide one potential, complex mechanism underlying eating disorders and explaining the partly shared etiopathogenesis of eating disorders and autoimmune diseases.

We observed an increased risk for autoimmune endocrinological and gastroenterological diseases among patients with eating disorders. Suggestive increases were found also for ocular diseases, dermatological and connective tissue diseases. These diseases originate from different organ systems, diverse HLA genotype and do not specifically cluster together. For example, the risk of T1D is related to HLADQ2/DQ8 genotype, whereas Crohn's disease does not show HLA association, but is associated with other immune regulation genes. Thus, the association of eating disorders with autoimmune diseases is not explained by shared HLA related risk but is likely to be explained by shared general immune disturbances. Supporting the view of the importance of immune-mediated pathways in the development of eating disorders, a suggestive association with NCAM2 gene, which encodes protein belonging to the immunoglobulin superfamily, was recently found in genome-wide association analysis of AN [40]. Further supporting the role of autoimmune processes in eating disorders, we also observed a higher prevalence of autoimmune diseases in men with BN compared to women; this is in line with greater load of vulnerabilities required in males in order to develop eating disorders [41].

The peak onset of T1D in Finland is in childhood and in adolescence [42], and its prevalence among patients was over four-fold at the start of the follow-up compared to control individuals. In previous studies of eating disorders in individuals with T1D, the association has been explained by weight gain with insulin treatment, and subsequent body dissatisfaction combined with a possibility to control body weight by modifying insulin dosages [43]. Additionally, management of diabetes with strict diet, associated hypoglycemic episodes with intense hunger, and dysregulation of gastrointestinal hormones are recognized [43]. As discussed above, the risk of T1D in eating disorders could be explained by shared immunologicial disturbances related to the altered gut microbiota and dysregulated immune tolerance. It is also possible that T1D related autoantibodies, such as glutamic acid decarboxylase, which are also found in neurological disorders, such as in stiff-man syndrome and in bipolar disorder [44], could have a role in eating disorders. Supporting this, having T1D has been shown to increase the risk for mood disorders [6].

In gastroenterological autoimmune diseases, we observed increased period prevalence at the start of the follow-up and increased incidence afterwards. The increase was mostly due to Crohn's disease, increases in celiac disease and ulcerative colitis did not reach statistical significance. Association between Crohn's disease/ulcerative colitis and eating disorders has previously been reported in individual patients [19]. It has been interpreted to base on shifts in energy balance, demands for attention on eating, and availability of unusual compensatory methods such as stoma or malabsorption. However, it is possible that pro-inflammatory cytokines activated in Crohn's disease could contribute to the development of eating disorders. Particularly interesting is TNF-α, a key cytokine in Crohn's disease, also known as cachexin due to its strong appetite suppressing and weight loss inducing effect. Interestingly, anecdotal evidence suggests positive effect of anti-TNF-α treatment in Crohn's disease on AN [45]. In celiac disease, both brain-reactive autoantibodies as well as high prevalence of mood disorders have been reported [3], [6].

We observed significantly elevated risk of SLE in patients with eating disorders. Its role is supported by the occurrence of brain-reactive autoantibodies, the increased risk of SLE in mood disorders [6], and the high rate of neuropsychiatric manifestations in the patients with SLE [46]. The autoantibodies in Sjögren's syndrome are also classified as brain-reactive, and the risk of mood disorders is shown increased [6]. Anecdotal reports of the co-occurrence of rheumatoid arthritis and AN have been published [47]. Unfortunately, our sample was too small to draw firm conclusions; the risk of Sjögren's syndrome, rheumatoid arthritis and dermatological diseases were marginally increased.

### Strengths and limitations

Strengths of the study include large patient cohort with eating disorders, specifically BED, which is rare in register studies due to its absence from diagnostic systems as an official diagnostic entity prior DSM-5. Further strengths are long follow-up, and prospectively collected register data.

Despite large sample, our study unfortunately lacked power to detect the potential differences between patients and controls in rare autoimmune diseases, and we observed several marginally significant differences. Eating disorder diagnoses in our study were admission diagnoses and did not cover co-morbidity or diagnostic cross-overs. We neither had data of the age of eating disorder onset. It would have also been intriguing to explore deeper the sex differences in autoimmune diseases, but due to small number of male patients we were unable to do so. Potential bias may arise from more active diagnosing of autoimmune diseases in the patients with eating disorders due to their treatment in special health care. However, no significant differences in the evolution of incidence rates of a composite of incident autoimmune diseases between the patients and controls emerged, and we also observed a higher period prevalence of autoimmune in patients before they had entered into the treatment. Second, majority of the autoimmune diseases studied are diagnosed in a special health care in Finland. The treatments in hospitals are heavily subsidized yielding low socioeconomic barriers. An important exception relates to autoimmune thyroiditis, which is relatively common in the population and often diagnosed and treated in primary care; its data cannot therefore be taken fully into account. Prevalences of other autoimmune diseases among controls were in line with commonly described population prevalences, which further suggest that detection bias between patients and control individuals does not explain our results.

### Conclusion

In conclusion, we observed the association between eating disorders and several autoimmune diseases with different genetic backgrounds. Our data support the findings from other studies indicating the role of immunological mechanisms at least in a subpopulation of patients with eating disorders. We recommend that clinicians treating patients with eating disorders consider the increased risk of autoimmune diseases and the possible role of autoimmune processes underlying these individuals' somatic and neuropsychiatric symptoms related to mood disturbances, anxiety and disordered eating. Future studies are needed to explore the risk of autoimmune diseases and the immunological mechanisms in individuals with various eating disorders and their family members.

## Supporting Information

Figure S1 **Incidence rates (1/1000) as a function of follow-up time with 95% confidence intervals for a composite of all autoimmune diseases.** Top: the whole study population; bottom, left: all patients with eating disorders; bottom, right: all control individuals.
(DOCX)

Table S1 **Categorization of autoimmune diseases and diagnostic codes for each revision (8, 9, 10) of** ***International Classification of Diseases*** **(ICD) as applied in the study.**
(DOCX)

Table S2 **Autoimmune disease status per each autoimmune disease at the end of the study period covering the time before, during and after the treatment for an eating disorder for patients with eating disorders and for their control individuals.** Odds Ratios (OR) are presented if meaningful analyses were possible.
(DOCX)
