# Supplementary material for: The Increased Risk for Autoimmune Diseases in Patients with Eating Disorders
Source: PLoS One. 2014 Aug 22;9(8):e104845. doi: 10.1371/journal.pone.0104845 (PMC4141740; doi:10.1371/journal.pone.0104845)
Supplement: Figure S1 — Incidence rates (1/1000) as a function of follow-up time with 95% confidence intervals for a composite of all autoimmune diseases. Top: the whole study population; bottom, left: all patients with eating disorders; bottom, right: all control individuals. (DOCX) [file pone.0104845.s001.docx]

**Supporting Information**

**Figure S1**. Incidence rates (1/1000) as a function of follow-up time with 95% confidence intervals for a composite of all autoimmune diseases. Top: the whole study population; bottom, left: all patients with eating disorders; bottom, right: all control individuals.

| 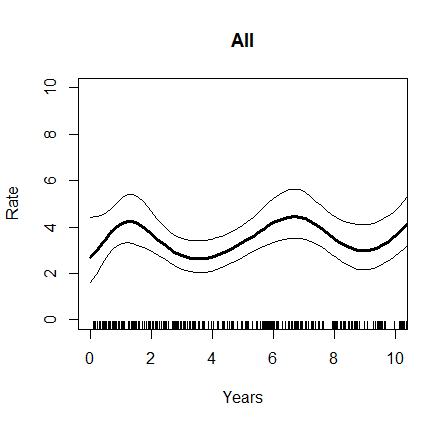 |  |
| --- | --- |
| 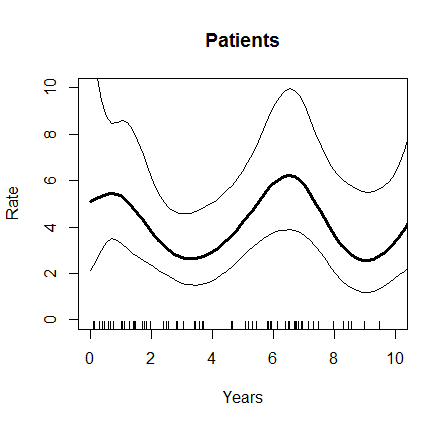 | 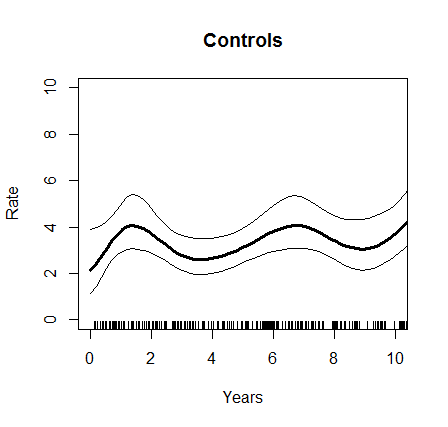 |
