# Supplementary material for: The Increased Risk for Autoimmune Diseases in Patients with Eating Disorders
Source: PLoS One. 2014 Aug 22;9(8):e104845. doi: 10.1371/journal.pone.0104845 (PMC4141740; doi:10.1371/journal.pone.0104845)
Supplement: Table S1 — Categorization of autoimmune diseases and diagnostic codes for each revision (8, 9, 10) of International Classification of Diseases (ICD) as applied in the study. (DOCX) [file pone.0104845.s002.docx]

**Supporting Information**

**Table S1.** Categorization of autoimmune diseases and diagnostic codes for each revision (8, 9, 10) of *International Classification of Diseases* (ICD) as applied in the study.

|  | | | |
| --- | --- | --- | --- |
| **Autoimmune disease** | **ICD-8** | **ICD-9** | **ICD-10** |
| ***Endocrinological diseases*** |  | | |
| Thyreotoxicosis (Basedow’s/Graves’ disease) | 242.00 | 242 | E05.0 |
| Autoimmune thyroiditis (Hashimoto's disease) | 245.03 | 2452A | E06.3 |
| Primary adrenocortical insufficiency (Addison’s disease) | 255.10 | 2554A | E27.1 |
| Diabetes type I | 250 | 2500B; 2501B; 2502B; 2503B; 2504B; 2505B; 2506B; 2507B; 2508B | E10 |
| ***Gastroenterological diseases*** |  | | |
| Celiac disease | 269.00 | 5790A | K90.0 |
| Regional enteritis (Crohn’s disease) | 563.00 | 555 | K50; M07.4 |
| Ulcerative colitis | 563.10 | 556 | K51; M07.5 |
| Primary biliary cirrhosis | 571.90 | 5716A | K74.3 |
| ***Ocular diseases*** |  | | |
| Iridocyclitis | 364 | 3640A; 3641A; 3642X; 3643X | H20 |
| ***Dermatological diseases*** |  | | |
| Pemphigus/pemphigoid | 694 | 6944A;6944B; 6944C; 6944D; 6945A; 6945B; 6946A | L10; L12 |
| Dermatitis herpetiformis | 693.99 | 6940A | L13.0 |
| Psoriasis | 696.00; 696.10; 696.19 | 6960A; 6961A; 6961B | L40; M07 |
| Vitiligo | 709.05 | 7090F | L80 |
| Lupus Erythematosus Discoides (LED) | 695.40 | 6954A | L93.0; G73.7; I32.8 |
| ***Connective tissue diseases*** |  | | |
| Rheumatoid arthritis | 712 | 714 | M05; M06; G73.7;I32.8; I39.8; I41.8; I52.8 |
| Athritis rheumatoides juvenilis | 712.00 | 7143A | M08 |
| Ankylosing spondylitis | 712.40 | 720 | M45; H22.1 |
| Polymyositis / Dermatomyositis | 716.10; 716.00 | 7104A; 7103B | M33 |
| Systemic lupus erythematosus (SLE) | 734.10 | 7100A | M32; G05.8; N16.4 |
| Systemic scleroderma | 734.00;734.01; 734.09 | 7101A | M34 |
| Mixed connective tissue disease (MCTD) | 734.91 | 7109X | M35.1; M35.8; M35.9 |
| Sjögren’s syndrome | 734.90 | 7102B | M35.0; G73.7 |
| Sarcoidosis | 135.99 | 1350A | D86; M14.8; G53.2; H22.1; I41.8; K77.8; M63.3; N16.2 |
| Vasculitides | 446; 287.00 | 4476A; 446; 6952C | M30, M31; M05.2; D69.0; I77.6; L95; M35.3; M35.6; M79.3; N08.5 |
| ***Neurological diseases*** |  | | |
| Multiple sclerosis | 340.99 | 340; 3400A | G35 |
| Myasthenia gravis | 733.00 | 3580A | G70.0 |
| ***Hematological diseases*** |  | | |
| Autoimmune hemolytic anemia | 283.90; 283.91 | 2830A; 2830B; 2830X; | D59 |
| Idiopathic thrombocytopenic purpura (ITP) | 287.10 | 2873A | D69.3 |
| Pernicious anemia | 281.00; 281.09 | 2810A; 2810X | D51 |
| ***Pulmonary diseases*** |  | | |
| Idiopathic fibrosing alveolitis | 517.01 | 5163A | J84 |
