# Supplementary material for: The Increased Risk for Autoimmune Diseases in Patients with Eating Disorders
Source: PLoS One. 2014 Aug 22;9(8):e104845. doi: 10.1371/journal.pone.0104845 (PMC4141740; doi:10.1371/journal.pone.0104845)
Supplement: Table S2 — Autoimmune disease status per each autoimmune disease at the end of the study period covering the time before, during and after the treatment for an eating disorder for patients with eating disorders and for their control individuals. Odds Ratios (OR) are presented if meaningful analyses were possible. (DOCX) [file pone.0104845.s003.docx]

**Supporting Information**

**Table S2.** Autoimmune disease status per each autoimmune disease at the end of the study period covering the time before, during and after the treatment for an eating disorder for patients with eating disorders and for their control individuals. Odds Ratios (OR) are presented if meaningful analyses were possible.

| **Autoimmune disease category** | **N (Positive/negative for the autoimmune disorder, % positive)** | | **OR (95% CI)** | **P-value** |
| --- | --- | --- | --- | --- |
|  | **Patients** | **Controls** |  |  |
| ***Endocrinological diseases*** | | | | |
| **Type I diabetes mellitus** | | | | |
| Anorexia Nervosa | 15/896 (1.6%) | 33/3611 (0.9%) | 1.86 (1.00-3.48) | 0.05 |
| Bulimia Nervosa | 47/1213 (3.7%) | 36/5004 (0.7%) | 5.74 (3.64-9.06) | <0.001 |
| Binge Eating Disorder | 6/165 (3.5%) | 7/677 (1.0%) | 3.43 (1.15-10.20) | 0.027 |
| All | 68/2274 (2.9%) | 76/9292 (0.8%) | 3.79 (2.70-5.31) | <0.001 |
| **Autoimmune hyperthyroidism (Basedow’s disease, Grave’s disease)** | | | | |
| Anorexia Nervosa | 5/906 (0.6%) | 21/3623 (0.6%) | 0.95 (0.35-2.57) | 0.92 |
| Bulimia Nervosa | 5/1255 (0.4%) | 31/5009 (0.6%) | 0.65 (0.25-1.66) | 0.36 |
| Binge Eating Disorder | 3/168 (1.8%) | 4/680 (0.6%) | 3.00 (0.67-13.40) | 0.15 |
| All | 13/2329 (0.6%) | 56/9312 (0.6%) | 0.93 (0.51-1.7) | 0.81 |
| **Autoimmune thyroiditis (Hashimoto’s disease)** | | | | |
| Anorexia Nervosa | 0/911 | 4/3640 (0.1%) | - | - |
| Bulimia Nervosa | 2/1258 (0.2%) | 5/5035 (0.1%) | - | - |
| Binge Eating Disorder | 0/171 | 0/684 | - | - |
| All | 2/2340 (0.09%) | 9/9359 (0.1%) | 0.89 (0.19-4.11) | 0.88 |
| **Adrenal insufficiency (Addison’s disease)** | | | | |
| Anorexia Nervosa | 1/910 (0.1%) | 0/3644 | *-* | *-* |
| Bulimia Nervosa | 0/1260 | 0/5040 | - | - |
| Binge Eating Disorder | 0/171 | 0/684 | - | - |
| All | 1/2341 (0.04%) | 0/9368 | - | - |
| ***Gastroenterological diseases*** | | | | |
| **Celiac disease** | | | | |
| Anorexia Nervosa | 6/905 (0.7%) | 20/3624 (0.5%) | 1.20 (0.48-3.00) | 0.70 |
| Bulimia Nervosa | 9/1251 (0.7%) | 22/5018 (0.4%) | 1.64 (0.75-3.55) | 0.21 |
| Binge Eating Disorder | 1/170 (0.6%) | 2/682 (0.3%) | 2.00 (0.18-22.06) | 0.57 |
| All | 16/2326 (0.7%) | 44/9324 (0.5%) | 1.45 (0.82-2.58) | 0.2 |
| **Regional enteritis (Crohn’s disease)** | | | | |
| Anorexia Nervosa | 10/901 (1.1%) | 8/3636 (0.2%) | 5.00 (1.97-12.67) | <0.001 |
| Bulimia Nervosa | 16/1244 (1.3%) | 26/5014 (0.5%) | 2.46 (1.32-4.59) | 0.005 |
| Binge Eating Disorder | 1/170 (0.6%) | 1/683 (0.1%) | 4.00 (0.25-63.95) | 0.33 |
| All | 27/2315 (1.1%) | 35/9333 (0.4%) | 3.09 (1.87-5.10) | <0.001 |
| **Ulcerative colitis** | | | | |
| Anorexia Nervosa | 5/906 (0.6%) | 21/3613 (0.6%) | 0.95 (0.36-2.55) | 0.92 |
| Bulimia Nervosa | 17/1243 (1.3%) | 46/4994 (0.9%) | 1.49 (0.85-2.61) | 0.17 |
| Binge Eating Disorder | 2/169 (1.2%) | 2/682 (0.2%) | 4.00 (0.56-28.40) | 0.17 |
| All | 24/2318 (1.0%) | 69/9299 (0.7%) | 1.4 (0.88-2.24) | 0.16 |
| **Primary biliary cirrhosis** | | |  | |
| Anorexia Nervosa | 0/911 | 0/3644 | *-* | *-* |
| Bulimia Nervosa | 0/1260 | 2/5038 (0.04%) | *-* | *-* |
| Binge Eating Disorder | 0/171 | 0/684 | - | - |
| All | 0/2342 | 2/9366 (0.02%) | - | - |
| ***Ocular diseases*** | | | | |
| **Iridocyclitis** | | | | |
| Anorexia Nervosa | 7/904 (0.8%) | 17/3627 (0.5%) | 1.65 (0.68-4.00) | 0.27 |
| Bulimia Nervosa | 11/1249 (0.9%) | 44/4996 (0.9%) | 1.00 (0.51-1.95) | 1.00 |
| Binge Eating Disorder | 6/165 (3.5%) | 2/682 (0.3%) | 12.0 (2.42-59.45) | 0.002 |
| All | 24/2318 (1.0%) | 63/9305 (0.7%) | 1.54 (0.96-2.47) | 0.08 |
| ***Dermatological diseases*** | | | | |
| **Pemphigus/pemphigoid** | | | | |
| Anorexia Nervosa | 0/911 | 1/3643 (0.02%) | *-* | *-* |
| Bulimia Nervosa | 0/1260 | 1/5039 (0.2%) | - | - |
| Binge Eating Disorder | 1/170 (0.6%) | 1/683 (0.1%) | - | - |
| All | 1/2341 (0.04%) | 3/9365 (0.03%) | 1.33 (0.14-12.8) | 0.8 |
| **Dermatitis herpetiformis (skin condition associated with celiac disease)** | | |  | |
| Anorexia Nervosa | 0/911 | 0/3644 | *-* | *-* |
| Bulimia Nervosa | 0/1260 | 0/5040 | - | - |
| Binge Eating Disorder | 0/171 | 0/684 | - | - |
| All | 0/2342 | 0/9368 | - | - |
| **Psoriasis** | | | | |
| Anorexia Nervosa | 4/907 (0.4%) | 15/3629 (0.4%) | 1.07 (0.35-3.21) | 0.91 |
| Bulimia Nervosa | 15/1245 (1.2%) | 38/5002 (0.8%) | 1.58 (0.87-2.87) | 0.13 |
| Binge Eating Disorder | 3/168 (1.8%) | 3/681 (0.4%) | 4.00 (0.81-19.82) | 0.09 |
| All | 22/2320 (0.9%) | 56/9312 (0.6%) | 1.57 (0.96-2.57) | 0.07 |
| **Vitiligo** | | | | |
| Anorexia Nervosa | 1/910 (0.1%) | 0/3644 | *-* | *-* |
| Bulimia Nervosa | 0/1260 | 3/5037 (0.06%) | - | - |
| Binge Eating Disorder | 0/171 | 0/684 | - | - |
| All | 1/2341 (0.04%) | 3/9365 (0.03%) | 1.33 (0.14-12.8) | 0.8 |
| **Lupus Erythematosus Discoides (LED)** | | | | |
| Anorexia Nervosa | 0/911 | 0/3644 | *-* | *-* |
| Bulimia Nervosa | 1/1259 (0.08%) | 1/5039 (0.02%) | - | - |
| Binge Eating Disorder | 0/171 | 0/684 | - | - |
| All | 1/2342 (0.04%) | 1/9367 (0.01%) | 4.0 (0.25-64.0) | 0.33 |
| ***Connective tissue diseases*** | | | | |
| **Rheumatoid arthritis** | | | | |
| Anorexia Nervosa | 6/905 (0.7%) | 10/3634 (0.3%) | 2.40 (0.87-6.60) | 0.09 |
| Bulimia Nervosa | 6/1254 (0.5%) | 28/5012 (0.6%) | 0.86 (0.36-2.07) | 0.73 |
| Binge Eating Disorder | 3/168 (1.8%) | 7/677 (1.0%) | 1.71 (0.44-6.63) | 0.43 |
| All | 15/2327 (0.6%) | 45/9323 (0.5%) | 1.33 (0.74-2.39) | 0.33 |
| Arthritis **rheumatoides juvenilis** | | | | |
| Anorexia Nervosa | 5/906 (0.6%) | 13/3631 (0.4%) | 1.54 (0.55-4.32) | 0.41 |
| Bulimia Nervosa | 3/1257 (0.2%) | 18/5022 (0.4%) | 0.67 (0.20-2.26) | 0.52 |
| Binge Eating Disorder | 3/168 (1.8%) | 3/681 (0.4%) | 4.00 (0.81-19.82) | 0.09 |
| All | 11/2331 (0.5%) | 34/9334 (0.4%) | 1.29 (0.66-2.55) | 0.46 |
| **Ankylosing spondylitis** | | | | |
| Anorexia Nervosa | 1/910 (0.1%) | 7/3637 (0.2%) | *-* | *-* |
| Bulimia Nervosa | 5/1255 (0.4%) | 17/5023 (0.3%) | - | - |
| Binge Eating Disorder | 1/170 (0.6%) | 6/678 (0.9%) | - | - |
| All | 7/2335 (0.3%) | 30/9338 (0.3%) | 0.93 (0.41-2.12) | 0.87 |
| **Polymyositis / Dermatomyositis** | | |  | |
| Anorexia Nervosa | 1/910 (0.1%) | 2/3642 (0.05%) | *-* | *-* |
| Bulimia Nervosa | 0/1260 | 1/5039 (0.02%) | - | - |
| Binge Eating Disorder | 0/171 | 1/683 (0.1%) | - | - |
| All | 1/2341 (0.04%) | 4/9364 (0.04%) | 1.0 (0.11-8.95) | 1.00 |
| **Systemic lupus erythematosus (SLE)** | | | | |
| Anorexia Nervosa | 1/910 (0.11%) | 1/3643 (0.03%) | *-* | *-* |
| Bulimia Nervosa | 2/1258 (0.16%) | 5/5035 (0.1%) | - | - |
| Binge Eating Disorder | 3/168 (1.75%) | 0/684 | - | - |
| All | 6/2336 (0.3%) | 6/9362 (0.06%) | 4.0 (1.29-12.4) | 0.02 |
| **Systemic scleroderma** | | | | |
| Anorexia Nervosa | 0/911 | 1/3643 (0.03%) | *-* | *-* |
| Bulimia Nervosa | 0/1260 | 0/5040 | - | - |
| Binge Eating Disorder | 1/170 (0.6%) | 1/683 (0.1%) | - | - |
| All | 1/2341 (0.04%) | 2/9366 (0.02%) | 2.0 (0.18-22.1) | 0.57 |
| **Mixed Connective Tissue Disease (MCTD)** | | | | |
| Anorexia Nervosa | 1/910 (0.1%) | 0/3644 | *-* | *-* |
| Bulimia Nervosa | 0/1260 | 1/5039 (0.02%) | - | - |
| Binge Eating Disorder | 1/170 (0.6%) | 1/683 (0.1%) | - | - |
| All | 2/2340 (0.09%) | 2/9366 (0.02%) | 4.0 (0.56-28.4) | 0.17 |
| **Sjögren’s syndrome** | | | | |
| Anorexia Nervosa | 3/908 (0.3%) | 2/3642 (0.05%) | *-* | *-* |
| Bulimia Nervosa | 4/1256 (0.3%) | 9/5031 (0.2%) | - | - |
| Binge Eating Disorder | 2/169 (1.2%) | 6/678 (0.9%) | - | - |
| All | 9/2333 (0.4%) | 17/9351 (0.2%) | 2.12 (0.94-4.75) | 0.07 |
| **Sarcoidosis** | | | | |
| Anorexia Nervosa | 0/911 | 0/3644 | *-* | *-* |
| Bulimia Nervosa | 0/1260 | 0/5040 | - | - |
| Binge Eating Disorder | 0/171 | 2/682 (0.3%) | - | - |
| All | 0/2342 | 2/9366 (0.02%) | - | - |
| Vasculitides | | | | |
| Anorexia Nervosa | 0/911 | 1/3643 (0.03%) | *-* | *-* |
| Bulimia Nervosa | 2/1258 (0.2%) | 1/5039 (0.02%) | - | - |
| Binge Eating Disorder | 0/171 | 2/682 (0.3%) | - | - |
| All | 2/2340 (0.09%) | 4/9364 (0.04%) | 2.0 (0.37-10.90) | 0.42 |
| ***Neurological diseases*** | | | | |
| **Multiple sclerosis** | | | | |
| Anorexia Nervosa | 5/906 (0.6%) | 9/3635 (0.2%) | *-* | *-* |
| Bulimia Nervosa | 3/1257 (0.2%) | 20/5020 (0.4%) | - | - |
| Binge Eating Disorder | 0/171 | 2/682 (0.3%) | - | - |
| All | 8/2334 (0.3%) | 31/9337 (0.3%) | 1.03 (0.48-2.25) | 0.94 |
| **Myasthenia gravis** | | | | |
| Anorexia Nervosa | 0/911 | 1/3643 (0.03%) | *-* | *-* |
| Bulimia Nervosa | 2/1258 (0.2%) | 4/5036 (0.08%) | - | - |
| Binge Eating Disorder | 0/171 | 0/684 | - | - |
| All | 2/2340 | 5/9363 (0.05%) | 1.6 (0.31-8.25) | 0.57 |
| ***Hematological diseases*** | | | | |
| **Autoimmune hemolytic anemia** | | | | |
| Anorexia Nervosa | 1/910 (0.11%) | 0/3644 | - | - |
| Bulimia Nervosa | 1/1259 (0.08%) | 1/5039 (0.02%) | - | - |
| Binge Eating Disorder | 0/171 | 0/684 | - | - |
| All | 2/2340 (0.09%) | 1/9367 (0.01%) | 8.0 (0.73-88.20) | 0.09 |
| **Idiopathic thrombocytopenic purpura (ITP)** | | | | |
| Anorexia Nervosa | 0/911 | 2/3642 (0.05%) | - | - |
| Bulimia Nervosa | 0/1260 | 4/5036 (0.08%) | - | - |
| Binge Eating Disorder | 0/171 | 0/684 | - | - |
| All | 0 | 6/9362 (0.06%) | - | - |
| **Pernicious anemia** | | | | |
| Anorexia Nervosa | 1/910 (0.11%) | 0/3644 | - | - |
| Bulimia Nervosa | 0/1260 | 3/5037 (0.06%) | - | - |
| Binge Eating Disorder | 0/171 | 0/684 | - | - |
| All | 1/2341 (0.04%) | 3/9365 (0.03%) | 1.33 (0.14-12.8) | 0.8 |
| ***Pulmonary diseases*** | | | | |
| **Idiopathic fibrosing alveolitis** | | | | |
| Anorexia Nervosa | 0/911 | 0/3644 | - | - |
| Bulimia Nervosa | 1/1259 (0.08%) | 0/5040 | - | - |
| Binge Eating Disorder | 0/171 | 0/684 | - | - |
| All | 1/2341 (0.04%) | 0/9368 | - | - |
